# Peristaltic channel flow and heat transfer of Carreau magneto hybrid nanofluid in the presence of homogeneous/heterogeneous reactions

**DOI:** 10.1038/s41598-020-68409-0

**Published:** 2020-07-13

**Authors:** Aneela Bibi, Hang Xu

**Affiliations:** State Key Lab of Ocean Engineering, School of Naval Architecture, Ocean and Civil Engineering, Shanghai Jiao Tong University, Shanghai, 200240 China

**Keywords:** Nanoscience and technology, Physics

## Abstract

The purpose of present work is to explore the features of homogeneous–heterogeneous reactions in peristalsis flow of Carreau magneto hybrid nanofluid with copper and silver nanoparticles in a symmetric channel. The velocity slip condition and thermal radiation effect is also taken in the simplified model. Thermodynamic optimization aspect is discussed through the entropy generation analysis. The proposed mathematical systems are modified by using a lubrication approach and solved by a homotopy-based package-BVPh 2.0. The impacts of different involved parameters on flow characteristics, thermal characteristics, chemically reactive concentration and entropy generation are scrutinized through analytic results. It reveals that the fluid velocity decreases with the increasing values of the Weissenberg and the Hartman numbers. Characteristics of the Brinkman and the thermal radiation numbers are quite reverse for the heat transfer rate. In addition, entropy generation decreases with thermal radiation and Weissenberg number. The main outcome signifies that hybrid nanofluid is better thermal conductor as compared to the conventional nanofluid.

## Introduction

There are growing interests on studies of peristalsis of non-Newtonian fluid flows due to their fundamental importance in physiological, engineering, industrial and medical applications. In biological process, such motion occurs in movement of chyme in gastrointestinal tract^1^, circulation of blood in arterioles^2^, passage of urine in ureters^3^, passage of ovum in the fallopian tube^4^, swallowing food via esophagus^5^ and so on. Important characteristics of peristalsis of viscous fluid is initially studied by Latham^6^ and Shapiro et al.^7^, they examined peristalsis of viscous fluid in channel/tube under lubrication approaches. In industrial process, peristaltic mechanism is used to control the fluids transport inside tracts. This mechanism is utilized for additional pumping of flow in hurt lung machine to prevent blockage and being fluid contents keep apart from tract boundaries. On the other hand, the concept of magnetohydrodynamics (MHD) has gained a lot of attention due its variety of applications in geophysics, astrophysics and engineering. It plays very important roles in design of nuclear reactors, satellite, gas turbines, missiles and the blood control during cardiac surgeries. Studies on peristalsis flows with MHD and other aspects have been investigated^8–14^.

The thermally efficient nanofluids, after dispersing nanomatrials such as silver, copper, aluminum and titanium etc., into base fluids such as water, ethylene glycol, propylene glycol and oil, was first discovered by Choi^15^. He noticed that, when the nanoparticles are dispersed into base fluid, the thermal capability of traditional heat transfer fluids will be enhanced significantly. Das et al.^16^ found that, when 1–4% aluminum nanoparticles are added into water, the thermal conductivity could increase by 10–25%. Due to intensive merits of nanofluids like high thermal conductivity at lower nanomaterials aggregation, stability for long term , minimum clogging in passages flow and homogeneousness they are expected to be utilized in different engineering and industrial areas, as well as in nanobiotechnology. Hamid et al.^17^ used the Crank Nicolson finite difference scheme to obtain the numerical solutions of inclined magnetohydrodynamics unsteady radiating flow in an open ended vertical channel with natural convection in the presence of nanoparticles. They revealed that with the increasing values of fractional parameter and for slighter values of time the velocity of the fluid increases, but the behavior is reverse after some critical values of time. Sheikholeslami et al.^18^ analyzed the solicitation of nanoparticle-enhanced phase change material in appearance of metallic fins and nanoparticles. They utilized the finite element method based on Galerkin to obtain the numerical results. They found enhancement in rate of solidification with increasing shape factor, even though there is a reduction in temperature. Several attempts of nanofluids under different aspects have been conducted^19–37^.

The new trend of the development of nanofluids is to manufacture hybrid nanofluids, which are basically in suspension of two or more nanoparicles into base fluid. Hybrid nanofluids overcome the harness, the synergic influence and flaws of individual suspension of nanoparticles, which are expected to hold better heat transfer rate and more remarkable thermal conductivity as compared with traditional nanofluids. Excellent characteristics of hybrid nanofluids make them very potential and valuable in various areas like electronic applications, lubrications, heat interchangers, space air-crafts, refrigeration of electronic apparatus, transportation industry, drug reduction and biomedical and so on. Several experimental and theoretical studies have been available on hybrid nanofluids. For example, Suresh et al.^38^ and Momin^39^ provided very useful experimental data for understanding the thermal performance of hybrid nanoparticles in various solutions. Xu and Sun^40^ investigated that the generalized hybrid nanofluid model for description of base fluids suspended with multiple kinds of solid particles in a vertical microchannel with mixed convection. Their model was then employed by Saqib et al.^41^ for investigation of natural convection flow of a hybrid nanofluid between two infinite vertical parallel plates. In literature survey, other recent investigations on hybrid nanofluids have been conducted by^42–53^.

Thermodynamic irreversibility occurring in a flow system provides intuition of losses related to the system. The major losses can be accounted due to heat transfer, friction, chemical reactions, explosion and mixing of fluids etc. Entropy generation is used to quantify these thermodynamic irreversibilities within a system. Moreover, entropy minimization provide information about quantification of irreversibilities and can be used to lower these losses in the flow system. The pioneer behind the discovery of entropy generation minimization (EGM) was Bejan^54^, who found that through entropy generation minimization the thermal efficiency of flow systems could be improved. His discovery opened the doors of new research eras for many researchers and scientists due to its remarkable performance in many manufacturing processes like turbo machinery, heat exchangers, electronic cooling devices and so on. Khan et al.^55^ modeled the problem between two stretchable rotating disks with gyrotactic microorganisms and entropy generation. Many entropy generation related studies have been done in different types of fluid systems^56–59^.

It is seen from literature that no attention has yet been paid for discussions of influences of chemical reactions on peristaltic flows with various configurations such as slip condition, MHD, thermal radiation and hybrid nanoparticles. Note that homogeneous and heterogeneous reaction are significant in various chemical reaction systems, for instance combustion, catalysis, electrochemical and biochemical systems and production of semiconductor films. Examples of such processes include coal gasification, iron production in blast furnaces and the oxidative regeneration in coked catalysis etc. Chaudhary and Merkin^60^ firstly proposed a homogeneous-heterogeneous reaction model, in which the homogeneous (bulk) reaction is assumed by cubic autocatalator kinetics and the heterogeneous (surface) reaction by a first order process. Other investigations on homogeneous/heterogeneous reactions with different aspects have been performed by^61–65^.

In this paper, the peristalsis transport of electrically conducting hybrid nanoparticles subject to thermal radiation and homogeneous-heterogeneous reactions are explored owing to its great potentials in cancer therapy and medicine production. In this analysis, two types of nanoparticles are taken, one are the silver nanoparticles used for the conventional nanofluid case and the other are the combination of silver and copper nanoparticles for hybrid nanofluid. Thermal radiation, slip effect and homogeneous/heterogeneous reactions are examined simultaneously. The multi-physical nonlinear systems are solved by a BVPH 2.0 package. Physical elucidation of analytic results is given with major findings being pointed out. Also, a comparison is made of the results of the hybrid suspension of those nanofluid with same nanoparticles volume fraction and pure water. Such model has great significance not only of its theoretical interest, but also in engineering applications.

## Problem statement

**Figure 1:**
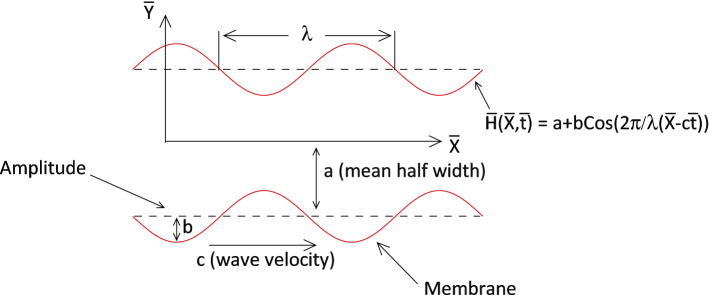
Physical sketch.

Consider a magnetohydrodynamic flow of an incompressible Carreau fluid in suspension either nanoparticles (silver) or hybrid nanoparticles (silver and copper) in a channel separated by a distance 2*a* driven by peristaltic motion of its symmetrical walls. The peristalsis of the channel waves is assumed to be the form of sinusoidal waves which move along the direction of channel length with a constant speed *c*. The physical sketch of this problem is shown in Fig. 1, in which ${\bar{X}}$- and ${\bar{Y}}$-axes are the Cartesian coordinates with ${\bar{X}}$ being along the channel flow direction and ${\bar{Y}}$ being normal to it. It is also assumed that the nanoparticles are distributed uniformly so that the agglomeration effect of nanomaterials is neglected. Other physical assumptions of the model are illustrated hereinafter.

The peristaltic structure of the channel walls is defined, based on the work of Hayat et al.^10^, as

1 $$\begin{aligned} {\bar{H}}\left( {\bar{X}},{\bar{t}} \right) =a+b\cos \left[ \frac{2\pi }{\lambda } \left( {\bar{X}}-c{\bar{t}}\right) \right] , \end{aligned}$$

where *a*, *b*,  $\lambda ,$ and ${\bar{t}}$, respectively, denote, the half width of the symmetric channel, the wave amplitude, the wave length and the time.

The generalized Ohm’s law for description of viscous incompressible electrically conducting fluid flow is written as

2 $$\begin{aligned} \mathbf {J}=\sigma \left( {\mathbf {E}}+\mathbf {V\times B}\right) , \end{aligned}$$

where ${\mathbf {J}}$ is the current density, $\sigma$ is the electrical conductivity, ${\mathbf {E}}$ is the electric field, ${\mathbf {V}}$ is the fluid velocity, ${\mathbf {B}}={\mathbf {b}}+\mathbf {B_0}$ is the total magnetic field where ${\mathbf {b}}$ is the induced magnetic field and ${\mathbf {B}}_0$ is the applied magnetic field. In magnetohydrodynamic flow studies, It is a common practice to neglect the induced magnetic field so that the total magnetic field ${\mathbf {B}}$ is approximately equal to the applied constant magnetic field ${\mathbf {B}}_0$. In our case, we assume it only distributes along ${\bar{Y}}$-axis and is governed by

3 $$\begin{aligned} {\mathbf {B}}_{0}=(0,B_{0},0). \end{aligned}$$

On the other hand, since there is no excess charge density, that implies $\nabla \cdot {\mathbf {E}}=0$. So Eq. (2) is reduced to

4 $$\begin{aligned} \mathbf {J}=\sigma \left( \mathbf {V}\times \mathbf {B}_{0}\right) . \end{aligned}$$

In a Carreau fluid, the constitutive equation for the extra stress tensor ${\bar{\mathbf {S}}}$ is described, refer to Hayat et al.^11^, as

5 $$\begin{aligned} {\bar{\mathbf {S}}}=\left\{ \eta _{\infty }+\left( \eta _{0}-\eta _{\infty }\right) \left[ 1+\left( \Gamma \,{\overline{\gamma }}\right) ^{2}\right] ^{ \frac{n-1}{2}}\right\} {\overline{\gamma }}, \end{aligned}$$

where $\eta _{\infty }$ and $\eta _{0}$ denote shear-rate viscosity at infinity and at the initial position respectively, $\Gamma$ is the material constant, *n* is the power-law index, and ${\overline{\gamma }}$ is the strain rate tensor defined by

6 $$\begin{aligned} {\overline{\gamma }}=\sqrt{\frac{1}{2}\underset{i}{\Sigma }\underset{j}{ \Sigma }{\overline{\gamma }}_{ij}{\overline{\gamma }}_{ji}}=\sqrt{ \frac{1}{2}{{\bar{\Lambda }}}}, \end{aligned}$$

with ${\bar{\Lambda }}$ representing the second invariant strain tensor defined by ${\bar{\Lambda }}=\text{ tr }[\text{ grad }{\bar{\mathbf {V}}}+(\text{ grad }{\bar{\mathbf {V}}} )^{T}]^{2}$. Here we consider the special case $\eta _{\infty }=0$ and $\Gamma {\overline{\gamma }}<1$, as a result ${\bar{\mathbf {S}}}$ is reduced to

7 $$\begin{aligned} \bar{\mathbf {S}}=\eta _{0}\left[ 1+\left( \Gamma {\overline{\gamma }} \right) ^{2}\right] ^{\frac{n-1}{2}}{\overline{\gamma }}. \end{aligned}$$

Note that the above correlation recovers a viscous material when $\Gamma =0$.

The radiative heat flux $q_{r}$ is taken along ${\bar{Y}}$-direction in thermal energy conservation equation, which is simplified, by means of the Rossland assumption^12^ as

8 $$\begin{aligned} q_{r}=-\frac{4\sigma ^{*}}{3k^{*}}\frac{\partial {\bar{T}}^4}{ \partial {\bar{Y}}}, \end{aligned}$$

where $\sigma ^{*}$ is the Stefan-Boltzmann constant with its value being $1.380648\times 10^{-3}$ and $k^{*}$ is the mean absorption coefficient. Note that the thickness of the thermal boundary layer is far thinner than the whole fluid domain, as a result, we are able to assume a linear relationship between the temperature of the thermal boundary layer ${\bar{T}}$ and the temperature of the free stream temperature $T_1$, which is expanded, by omitting higher-order terms, by

9 $$\begin{aligned} {\bar{T}}^{4}=4{T}_{1}^{3}{\bar{T}}-3{T}_{1}^{4}. \end{aligned}$$

Substituting Eq. (9) into Eq. (8), we obtain

10 $$\begin{aligned} \frac{\partial q_{r}}{\partial {\bar{Y}}}=-\frac{16 T_{1}^{3}\sigma ^{*}}{3k^{*}}\frac{\partial ^{2} {\bar{T}}}{\partial {\bar{Y}}^{2}}. \end{aligned}$$

The simple model for homogeneous and heterogeneous chemical reactions in the boundary layer flow^60^ is employed, in which the isothermal cubic autocatalysis of the homogeneous reaction occurs in the bulk, governed by

$$\begin{aligned} A+2B\rightarrow 3B,\quad \text{ rate }=k_{c}{\bar{\alpha }} \bar{\beta ^{2}}, \end{aligned}$$

while the first order isothermal heterogeneous reaction takes place on the catalyst surface, given by

$$\begin{aligned} A\rightarrow B, \quad \text{ rate }=k_{s}{\bar{\alpha }}, \end{aligned}$$

where ${\bar{\alpha }}$ and ${\bar{\beta }}$ are concentrations of the species *A* and *B* respectively, $k_{c}$ the homogeneous reaction coefficient, $k_{s}$ the heterogeneous reaction coefficient. It is assumed that there is only the homogeneous reaction in the bulk so that the reaction rate for the heterogeneous reaction in the outer flow is zero.

**Table 1:** Thermo-physical properties of nanofluid^19^ and hybrid nanofluid^40^.

Property	Type	Correlation
Viscosity	I	$\mu _{_{nf}}=\frac{\mu _{_{f}}}{(1-\phi )^{2.5}}$
	II	$\mu _{_{hnf}}=\frac{\mu _{_{{f}}}}{(1-\phi _{Ag}-\phi _{Cu})^{2.5}}$
Density	I	$\rho _{_{nf}}=(1-\phi )\rho _{_{f}}+\phi \rho _{_{s}}$
	II	$\rho _{_{hnf}}=(1-\phi _{Ag}-\phi _{Cu})\rho _{_{f}}+\phi _{_{Ag}}\rho _{_{Ag}}+\phi _{_{Cu}}\rho _{_{Cu}}$
Heat capacity	I	$(\rho c_{_p})_{_{nf}}=(1-\phi )(\rho c_{_p})_{_{f}}+\phi (\rho c_{_p})_{_s}$
	II	$(\rho c_{_p})_{_{hnf}}=(1-\phi _{Ag}-\phi _{Cu})(\rho c_{p})_{f}+\phi (\rho c_{p})_{Ag}+\phi (\rho c_{p})_{Cu}$
Electric conductivity	I	$\sigma _{nf}=\frac{\sigma _{s}(1+2\phi )+2\sigma _{_{f}}(1-\phi )}{\sigma _{_{s}}(1-\phi ) +\sigma _{_{f}}(2+\phi )}\sigma _{_{f}}$
	II	$\sigma _{_{hnf}}= \frac{\sigma _{Cu}(1+2\phi _{Cu})+2\sigma _{_{bf}}(1-\phi _{Cu})}{\sigma _{Cu}(1-\phi _{Cu})+\sigma _{_{bf}} (2+\phi _{Cu})}\sigma _{_{bf}}$, where
		$$\sigma _{_{bf}} =\frac{\sigma _{Ag}(1+2\phi _{Ag})+2\sigma _{_{f}}(1-\phi _{Ag})}{\sigma _{Ag}(1-\phi _{Ag})+\sigma _{_{f}}(2+\phi _{Ag})}\sigma _{_{f}}$$
Thermal conductivity	I	$k_{nf}=\frac{k_{s}+2k_{_{}f}-2\phi (k_{_{}f}-k_{s})}{k_{s}+2k_{f}+\phi (k_{f}-k_{s})}k_{_{f}}$
	II	$k_{_{hnf}}=\frac{k_{{Cu}}+2 k_{_{bf}}-2\phi _{Cu}(k_{_{bf}}-k_{{Cu}})}{k_{{Cu}}+2k_{_{bf}}+\phi _{Cu}(k_{_{bf}}-k_{{Cu}})}k_{_{bf}}$, where
		$$k_{_{bf}} =\frac{k_{_{Ag}}+2 k_{_{f}}-2\phi _{Ag}(k_{_{f}}-k_{_{Ag}})}{k_{_{Ag}}+2k_{_{f}}+\phi _{Ag}(k_{_{f}}-k_{_{Ag}})}k_{_{f}}$$

It has known that the nanofluid refers to a base fluid in suspension of solid particles in nanoscale, whose thermo-physical properties can be obtained using the following correlations denoted in Table 1. Here I and II denote conventional nanofluid and hybrid nanofluid respectively, $\phi$ is the nanoparticles volume fraction with *Ag* representing the silver and *Cu* representing the Copper, $\rho$, $\mu$, $c_p$ and *k* are, respectively, the density, viscosity, specific heat capacity, thermal conductivity, the subscripts *s*, *f*, *nf* and *hnf* denote the nanoparticles, the base fluid, the nanofluid and the hybrid nanofluid respectively.

**Figure 2:**
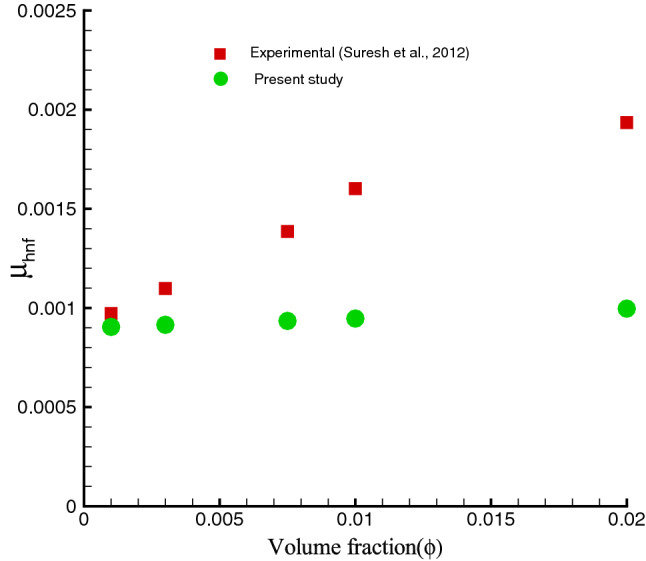
Viscosity of Ag–Cu/water hybrid nanofluid.

**Figure 3:**
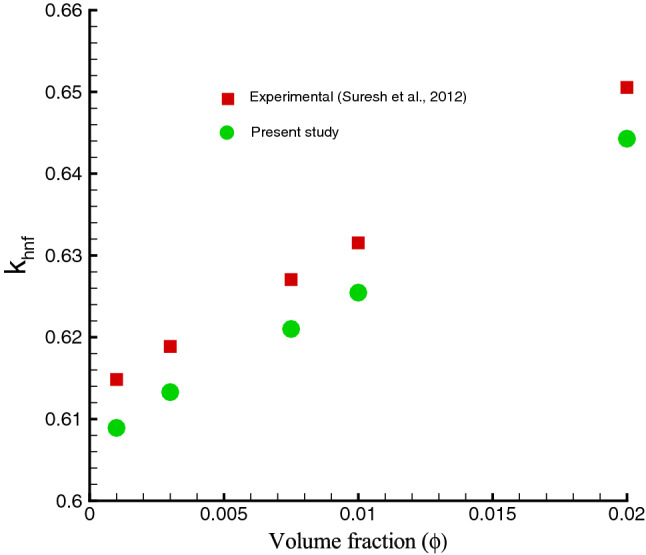
Thermal conductivity of Ag–Cu/water hybrid nanofluid.

It should be pointed out that the effective thermophysical properties of a hybrid nanofluid can be abridged to a regular nanofluid when either $\phi _{Ag}=0$ or $\phi _{Cu}=0$ is set to zero. The thermophysical properties of water at $25^{o}C$ and solid nanomaterials are provided in Table 2. Note that the accuracy and validity of the mathematical models has been experimentally checked by by Suresh et al.^38^ for hybrid nanofluids, as shown in Figs. 2 and 3.

**Table 2:** Thermo-physical properties of water and solid nanoamaterials^45^.

Properties	$$ H_{2}O$$	*Ag*	*Cu*
$\beta (K^{-1})$	21	1.89	1.67
$\sigma (Sm^{-1})$	0.05	6.3	5.96
$\rho (kg m^{-3})$	997.0	10500	8933
$c_{p} (Jkg^{-1}K^{-1})$	4179	235	385
$k (Wm^{-1}K^{-1})$	0.613	429	400

## Governing equations

Under the above-mentioned assumptions, the governing equations embodying the conservations of the total mass, momentum, thermal energy and chemical diffusion are written as^63^

11 $$\begin{aligned} \frac{\partial {\bar{U}}}{\partial {\bar{X}}}+\frac{\partial {\bar{V}}}{\partial {\bar{Y}}}= & {} 0, \end{aligned}$$

12 $$\begin{aligned} \rho \frac{d {\bar{U}}}{d {\bar{t}}}= & {} -\frac{\partial {\bar{P}}}{\partial {\bar{X}}}+ \frac{\partial {\bar{S}}_{{\bar{X}}{\bar{X}}}}{\partial {\bar{X}}}+\frac{\partial {\bar{S}}_{{\bar{X}}{\bar{Y}}}}{\partial {\bar{Y}}}-\sigma B_{0}^{2}{\bar{U}}, \end{aligned}$$

13 $$\begin{aligned} \rho \frac{d {\bar{V}}}{d {\bar{t}}}= & {} -\frac{\partial {\bar{P}}}{\partial {\bar{Y}}}+ \frac{\partial {\bar{S}}_{{\bar{Y}}{\bar{X}}}}{\partial {\bar{X}}}+\frac{\partial {\bar{S}}_{{\bar{Y}}{\bar{Y}}}}{\partial {\bar{Y}}}, \end{aligned}$$

14 $$\begin{aligned} (\rho c_{p}) \frac{d {\bar{T}}}{d {\bar{t}}}= & {} k \left( \frac{\partial ^2 {\bar{T}}}{\partial {\bar{X}}^2}+\frac{\partial ^2 {\bar{T}}}{\partial {\bar{Y}}^2}\right) -\frac{\partial q_{r} }{\partial {\bar{Y}}} +\sigma B_{0}^{2}{\bar{U}}^{2} \nonumber \\&+\frac{\partial {\bar{U}}}{\partial {\bar{X}}}S_{{\bar{X}}{\bar{X}}}+\frac{\partial {\bar{V}}}{\partial {\bar{Y}}}S_{{\bar{Y}}{\bar{Y}}}+\left( \frac{\partial {\bar{U}}}{\partial {\bar{Y}}}+\frac{\partial {\bar{V}}}{\partial {\bar{X}}}\right) S_{{\bar{X}}{\bar{Y}}} , \end{aligned}$$

15 $$\begin{aligned} \frac{d {\bar{\alpha }}}{d {\bar{t}}}= & {} D_{A}\left( \frac{\partial ^2 {\bar{\alpha }}}{\partial {\bar{X}}^2}+\frac{\partial ^2 {\bar{\alpha }}}{\partial {\bar{Y}}^2}\right) -k_{c}{\bar{\alpha }} {\bar{\beta }}^{2}, \end{aligned}$$

16 $$\begin{aligned} \frac{d {\bar{\beta }}}{d {\bar{t}}}= & {} D_{B}\left( \frac{\partial ^2 {\bar{\beta }}}{\partial {\bar{X}}^2}+\frac{\partial ^2 {\bar{\beta }}}{\partial {\bar{Y}}^2}\right) +k_{c}{\bar{\alpha }}{\bar{\beta }}^{2}, \end{aligned}$$

in which ${d}/{d {\bar{t}}}=\left( {\partial }/{\partial {\bar{t}}}+{\bar{U}}{\partial }/{\partial {\bar{X}}}+{\bar{V}}{\partial }/{\partial {\bar{Y}}}\right)$ denotes the matrial time derivative, ${\bar{P}}$ the pressure, ${\bar{S}}_{{\bar{X}}{\bar{X}}}$, ${\bar{S}}_{{\bar{X}}{\bar{Y}}}$, ${\bar{S}}_{{\bar{Y}}{\bar{Y}}}$ are the extra stress tensor components, $D_{A}$ and $D_{B}$ are the diffusion coefficients of chemical species ${\bar{\alpha }}$ and ${\bar{\beta }}$ respectively.

The corresponding boundary conditions for this flow problem are given as:

17 $$\begin{aligned} {\bar{Y}}= & {} 0:\;\; \frac{\partial {\bar{U}}}{\partial {\bar{Y}}}=0,\;\; {\bar{T}} = T_{0}, \;\; {\bar{\alpha }} ={\alpha }_{0},\;\; {\bar{\beta }}=0; \nonumber \\ {\bar{Y}}= & {} H:\;\; {\bar{U}}+{\bar{\omega }}{\bar{S}}_{{\bar{X}}{\bar{Y}}}=0, \;\; \frac{\partial {\bar{T}}}{\partial {\bar{Y}}} =0,\;\; D_{A}\frac{\partial {\bar{\alpha }}}{\partial {\bar{Y}}} =-D_B \frac{\partial {\bar{\beta }}}{\partial {\bar{Y}}}=k_{s}{\bar{\alpha }}. \end{aligned}$$

Here ${\bar{\omega }}$ represents the velocity slip parameter at wall.

Define the traveling wave transformations

18 $$\begin{aligned} {\bar{x}}= & {} {\bar{X}}-c{\bar{t}},\text { }{\bar{y}}={\bar{Y}},\text { }{\bar{u}}({\bar{x}}, {\bar{y}})={\bar{U}}({\bar{X}},{\bar{Y}},{\bar{t}})-c,\text { }{\bar{v}}({\bar{x}},{\bar{y}})= {\bar{V}}({\bar{X}},{\bar{Y}},{\bar{t}}), \nonumber \\ {\bar{T}}({\bar{x}},{\bar{y}})= & {} {\bar{T}}({\bar{X}},{\bar{Y}},{\bar{t}}),\text { }{\bar{p}} \left( {\bar{x}},{\bar{y}}\right) ={\bar{P}}\left( {\bar{X}},{\bar{Y}},{\bar{t}}\right) . \end{aligned}$$

Then define the following dimensionless scaling transformations

19 $$\begin{aligned} x= & {} \frac{{\bar{x}}}{\lambda },\quad y=\frac{{\bar{y}}}{a},\quad t=\frac{c\, {\bar{t}}}{\lambda }, \quad h=\frac{{\bar{H}}}{a}, \quad u=\frac{{\bar{u}}}{c},\quad v=\frac{{\bar{v}}}{c\,\delta }, \nonumber \\ \gamma= & {} \frac{a\,{\bar{\gamma }}}{c}, \quad p=\frac{a^{2}{\bar{p}}}{c\,\eta _{0}\lambda }, \quad \omega =\frac{{\bar{\omega }}}{a}, \quad d =\frac{b}{a}, \quad f=\frac{{\bar{\alpha }}}{\alpha _{0}}, \quad g=\frac{{\bar{\beta }}}{\alpha _{0}},\nonumber \\ S _{xx}= & {} \frac{\lambda }{c\,\eta _{0}}{{\bar{S}}}_{\bar{x }{\bar{x}}},\quad S _{xy}=\frac{a}{c\,\eta _{0}}{{\bar{S}}}_{{\bar{x}}{\bar{y}}}, \quad S _{yy}\mathbf =\frac{a}{c\,\eta _{0}}{{\bar{S}}}_{{\bar{y}}{\bar{y}}}, \quad \theta =\frac{{\bar{T}}-T_{0}}{T_{1}-T_{0}} \end{aligned}$$

and the stream function $\psi$ by definition^10^ as

20 $$\begin{aligned} u=\frac{\partial \psi }{\partial y},\ v=-\delta \frac{\partial \psi }{\partial x}, \end{aligned}$$

in which $\delta ={a}/{\lambda }$ is the scaling wave number.

Substituting the transformations (18) and (19) into the governing Eqs. (11)–(16), the continuity equation (11) is automatically satisfied, other equations are reduced to

21 $$\begin{aligned} \delta {Re}A_{1}\left( \frac{\partial \psi }{\partial y}\frac{\partial }{ \partial x}-\frac{\partial \psi }{\partial x}\frac{\partial }{\partial y} \right) \frac{\partial \psi }{\partial y}= & {} -\frac{\partial p}{\partial x} +\delta ^{2}\frac{\partial S_{xx}}{\partial x}+\frac{\partial S_{xy}}{ \partial y}-M^{2}A_{2}\left( \frac{\partial \psi }{\partial y} +1\right) , \end{aligned}$$

22 $$\begin{aligned} -\delta ^{3}{Re}A_{1}\left( \frac{\partial \psi }{\partial y}\frac{\partial }{\partial x}-\frac{\partial \psi }{\partial x}\frac{\partial }{\partial y} \right) \frac{\partial \psi }{\partial x}= & {} -\frac{\partial p}{\partial y} +\delta ^{2}\frac{\partial S_{yx}}{\partial x}+\delta \frac{\partial S_{yy}}{ \partial y}, \end{aligned}$$

23 $$\begin{aligned} \delta {Re}\Pr A_{3}\left( \frac{\partial \psi }{\partial y}\frac{\partial }{ \partial x}-\frac{\partial \psi }{\partial x}\frac{\partial }{\partial y} \right) \theta & =  A_{4}\left( \delta ^{2}\frac{\partial ^{2}\theta }{\partial x^{2}}+ \frac{\partial ^{2}\theta }{\partial y^{2}}\right) +BrM^{2}A_{2} \left( \frac{\partial \psi }{\partial y}+1\right) ^{2} \nonumber \\ & \quad +Br\left[ \delta \frac{\partial ^{2}\psi }{\partial x\partial y} \left( S_{xx}-S_{yy}\right) +\left( \frac{\partial ^{2}\psi }{\partial y^{2}}-\delta ^{2}\frac{\partial ^{2}\psi }{\partial x^{2}}\right) S_{xy}\right] +R_{1}\frac{\partial ^{2}\theta }{\partial y^{2}}, \end{aligned}$$

24 $$\begin{aligned} \delta {Re}\left( \frac{\partial \psi }{\partial y}\frac{\partial }{ \partial x}-\frac{\partial \psi }{\partial x}\frac{\partial }{\partial y} \right) f= & {} \frac{1}{Sc}\left( \delta ^{2}\frac{\partial ^{2}f}{\partial x^{2}}-\frac{\partial ^{2}f}{\partial y^{2}}\right) -Kfg^{2}, \end{aligned}$$

25 $$\begin{aligned} \delta {Re}\left( \frac{\partial \psi }{\partial y}\frac{\partial }{ \partial x}-\frac{\partial \psi }{\partial x}\frac{\partial }{\partial y} \right) g= & {} \frac{\epsilon }{Sc}\left( \delta ^{2}\frac{ \partial ^{2}g}{\partial x^{2}}-\frac{\partial ^{2}g}{\partial y^{2}}\right) +Kfg^{2}, \end{aligned}$$

in which *Re*, *M*, *Pr*, *Br*, $R_1$, *Sc*, *K*, $\upsilon$ and $\epsilon$ are, respectively, the Reynold number, the Hartman number, the Prandtl number, the Brinkman number, the radiation parameter, the Schmidt number, the homogenous reaction ratio, kinematic viscosity and the ratio of diffusion coefficients. They are defined by

26 $$\begin{aligned} Re= & {} \frac{\rho \,c\,a}{\eta _{0}}, \;\; M =B_{0}\,a\sqrt{\frac{\sigma }{\eta _{0}} },\;\; Pr =\frac{\eta _{0}\,c_{p} }{k},\;\;Br=\frac{\eta _0c^{2}}{k(T_{1}-T_{0})}, \nonumber \\ R_{1}= & {} \frac{16 T_{1}^{3}\sigma ^{*}}{3kk^{*}}, \;\; Sc=\frac{\eta _{0}}{\rho D_{A}}, \;\; K=\frac{a^{2}k_{c}\alpha _{0}^{2}}{\upsilon },\;\; \upsilon =\frac{\eta _{0}}{\rho }, \;\; \epsilon =\frac{D_{B}}{D_{A}}. \end{aligned}$$

The constants $A_{1}$ to $A_{4}$ denoted in above equations are defined for the conventional nanofluid as

27 $$\begin{aligned} A_{1}=(1-\phi )+\frac{\rho _{s}\phi }{\rho _{f}}, \quad A_{2}=\frac{\sigma _{nf}}{\sigma _{f}}, \quad A_{3}=(1-\phi )+\frac{(\rho c_{p})_{s}}{(\rho c_{p})_{f}}, \quad A_{4}=\frac{k_{nf}}{k_{f}}, \end{aligned}$$

and for the hybrid nanofluid as

28 $$\begin{aligned} A_{1}= & {} \left[ (1-\phi _{Ag}-\phi _{Cu})+\frac{\phi _{Ag}\rho _{Ag}}{\rho _{f}} +\frac{\phi _{Cu}\rho _{Cu}}{\rho _{f}}\right] , \quad A_{2}=\frac{\sigma _{hnf}}{\sigma _{f}}, \nonumber \\ A_{3}= & {} \left[ (1-\phi _{Ag}-\phi _{Cu})+\frac{(\rho c_{p})_{Ag}}{(\rho c_{p})_{f}}+\frac{(\rho c_{p})_{Cu}}{(\rho c_{p})_{f}}\right] , \quad A_{4}=\frac{k_{hnf}}{k_{f}}. \end{aligned}$$

Also, the extra stress tensor components ($S_{xx},$ $S_{xy},$ $S_{yy}$) in above equations are defined, based on Eq. (5), as

29 $$\begin{aligned} S_{xx}= & {} 2\left( 1+\frac{n-1}{2}W_e^{2}{\gamma }^{2}\right) \frac{\partial ^{2}\psi }{\partial x\partial y}, \quad S_{yy} = 2\delta \left( 1+\frac{n-1}{2}W_e^{2}{\gamma }^{2}\right) \frac{ \partial ^{2}\psi }{\partial x\partial y}, \nonumber \\ S_{xy}= & {} \left( 1+\frac{n-1}{2}W_e^{2}{\gamma }^{2}\right) \left( \frac{ \partial ^{2}\psi }{\partial y^{2}}-\delta ^{2}\frac{\partial ^{2}\psi }{ \partial x^{2}}\right) , \end{aligned}$$

where $W_e={\Gamma c}/{a}$ denotes the Weissenberg number and ${\gamma }$ is given by the relation

30 $$\begin{aligned} {\gamma }=\sqrt{4\delta ^{2}\frac{\partial ^{2}\psi }{\partial x\partial y }+\left( \frac{\partial ^{2}\psi }{\partial y^{2}}-\delta ^{2}\frac{\partial ^{2}\psi }{\partial x^{2}}\right) ^{2}}. \end{aligned}$$

Under the assumptions of long wavelength ($\delta =a/\lambda$) and low Reynolds number, Eqs.(21) and (22) are written in the forms:

31 $$\begin{aligned}&-\frac{\partial p}{\partial x}+\frac{\partial }{\partial y}\left[ \left( 1+ \frac{n-1}{2}W_e^{2}\left( \frac{\partial ^{2}\psi }{\partial y^{2}}\right) ^{2}\right) \frac{\partial ^{2}\psi }{\partial y^{2}}\right] -M^{2}A_{2} \left( \frac{\partial \psi }{\partial y}+1\right) =0, \end{aligned}$$

32 $$\begin{aligned}&-\frac{\partial p}{\partial y}=0, \end{aligned}$$

which is reduced, by eliminating the pressure terms, to

33 $$\begin{aligned} \frac{\partial ^{2}}{\partial y^{2}}\left[ \left( 1+\frac{n-1}{2} W_e^{2}\left( \frac{\partial ^{2}\psi }{\partial y^{2}}\right) ^{2}\right) \frac{\partial ^{2}\psi }{\partial y^{2}}\right] -M^{2}A_{2} \frac{ \partial ^{2}\psi }{\partial y^{2}}=0. \end{aligned}$$

Similarly, Eq. (23) with a lubrication approach, gives

34 $$\begin{aligned} (A_{4}+R_{1})\frac{\partial ^{2}\theta }{\partial y^{2}}+Br\left( \frac{\partial ^{2}\psi }{\partial y^{2}}\right) ^{2}\left[ 1+\frac{n-1}{2}W_e^{2}\left( \frac{ \partial ^{2}\psi }{\partial y^{2}}\right) ^{2}\right] +BrM^{2}A_{2} \left( 1+ \frac{\partial \psi }{\partial y}\right) ^{2}=0. \end{aligned}$$

The chemically reactive homogeneous/heterogeneous equations are

35 $$\begin{aligned}&\frac{1}{Sc}\frac{\partial ^{2}f}{\partial y^{2}}-Kfg^{2}=0, \end{aligned}$$

36 $$\begin{aligned}&\frac{\epsilon }{Sc}\frac{\partial ^{2}g}{\partial y^{2}}+Kfg^{2}=0. \end{aligned}$$

For the special case $D_{A}=D_{B}$ ($\epsilon =1$), it holds $g=1-f$. As a result, Eqs.(35) and (36) are combined into

37 $$\begin{aligned} \frac{1}{Sc}\frac{\partial ^{2}f}{\partial y^{2}}-Kf\left( 1-f\right) ^{2}=0. \end{aligned}$$

The corresponding non-dimensional boundary conditions in wave frame are

38 $$\begin{aligned} \psi= & {} 0,\text { }\frac{\partial ^{2}\psi }{\partial y^{2}}=0,\text { at } y=0,\text { }\psi =F,\text { }\frac{\partial \psi }{\partial y}=-1-\omega S_{xy}\text { at }y=h, \nonumber \\ \theta= & {} 0\text { at }y=0,\frac{\partial \theta }{\partial y} =0\text { at } y=h, \nonumber \\ f= & {} 1\text { at }y=0,\text { }\frac{\partial f}{\partial y}=K_{s}f\text { at }y=h, \end{aligned}$$

in which $K_{s}={k_{s}a}/{D_{A}}$ is the heterogeneous reaction parameter, $h=1+d\cos 2\pi x$ is the dimensional shape of peristaltic wall, $\omega$ is the the velocity slip parameter and *F* the dimensionless time-mean flow in wave frame^7^ is

39 $$\begin{aligned} \Theta =F+1, \end{aligned}$$

where $\Theta$ the dimensionless flow rate in the laboratory frame and

40 $$\begin{aligned} F=\int _{0}^{h(x)}\frac{\partial \psi }{\partial y}dy=\psi (h)-\psi (0). \end{aligned}$$

## Verification of results

The BVPh 2.0 Mathematica package based on the optimal homotopy analysis method (OHAM) is applied to give solutions to the nonlinear linear system (33), (34) and (37) to get an accurate analytic solution. The BVPh 2.0 package is easy to use and free available online on (http://numericaltank.sjtu.edu.cn/BVPh2.0) with a user’s guide line. It provides the best solutions and it has been proved that its solution is close to the exact solution. Because HAM has some advantages over other traditional analytic approximation methods. First, HAM is independent of small/large physical parameters as compare to perturbation techniques and is valid in more general cases. Besides, it provides a convenient way to guarantee the convergence of series solution that is different from all other analytic techniques. Furthermore, if it is required to get solutions in polynomials, exponential or in trigonometric forms, then accordingly the base function is adjusted. Here, it is very convenient to use the power series as initial guesses since the boundary conditions fall in a finite domain [0, h]. So, the initial guesses are chosen as

41 $$\begin{aligned} {\psi _{0}}(y)= \frac{(3F+h)(1+\omega S_{xy})}{2h} y- \frac{(F+h)(1+\omega S_{xy})}{2h^3} y^3, \;\; {\theta _{0}}(y)=0, \;\;\; {f_{0}}(y)=\frac{K_{s}}{1+hK_{s}}y. \end{aligned}$$

Based on the boundary conditions (38) and the solution expressions, the linear operators are chosen as

42 $$\begin{aligned} L_{\psi }=\frac{\partial ^{4}}{\partial y^{4}},\;\;\; L_{\theta }=\frac{\partial ^{2}}{\partial y^{2}}, \;\;\; L_{f}=\frac{\partial ^{2}}{\partial y^{2}}. \end{aligned}$$

In the BVPh 2.0 technique, the convergence-control parameters $c_{01}$, $c_{02}$ and $c_{03}$ are determined through the minimum variance integral method^21^ and the optimal values of convergence-control parameters are usually found by minimizing the average squared residuals. In doing so, the direct command **GetOptiVar** is used for the values of convergence-control parameters. The approximated values of $c_{01}$, $c_{02}$ and $c_{03}$ are given in Table 3 where the corresponding values of all physical parameters are prescribed by $d=0.4$, $x=0.1$, $\Theta = 1.2$, $W_e=0.05$, $\omega =0.03$, $n=0.2$, $R_{1}=0.3$, $Br=1$, $M=0.5$, $\phi =\phi _{hnf}=0.05$, $Sc=0.5$, $K_{s}=0.5$ and $K=0.2$.

**Table 3:** Optimal values for convergence control parameters.

Volume fraction	$c_{01}$	$c_{02}$	$c_{03}$
(nanofluid) $\phi =0.05$	$-$ 0.989166	$-$ 0.684494	$-$ 0.176875
(hybrid nanofluid) $\phi _{Ag}+\phi _{Cu}=0.05$	$-$ 0.987589	$-$ 0.683218	$-$ 0.176875

To check the accuracy of our computational results, we define the following absolute error evaluating function based on Eqs. (33), (34) and (37) as

43 $$\begin{aligned} Err(N)=\max {[Err_{\psi }(N), Err_{\theta }(N), Err_f(N)]}, \end{aligned}$$

where

44 $$\begin{aligned} Err_{\psi }(N)= & {} \int _{0}^h\left\{ \frac{\partial ^{2}}{\partial y^{2}}\left[ \left( 1+\frac{n-1}{2} We^{2}\left( \frac{\partial ^{2}\psi }{\partial y^{2}}\right) ^{2}\right) \frac{\partial ^{2}\psi }{\partial y^{2}}\right] -M^{2}A_{2} \frac{\partial ^{2}\psi }{\partial y^{2}}\right\} ^{2}dy, \end{aligned}$$

45 $$\begin{aligned} Err_{\theta }(N)= & {} \int _{0}^h \bigg \{(A_{4}+R_{1})\frac{\partial ^{2}\theta }{\partial y^{2}}+Br\left( \frac{\partial ^{2}\psi }{\partial y^{2}}\right) ^{2}\left[ 1+\frac{n-1}{2}W_e^{2}\left( \frac{\partial ^{2}\psi }{\partial y^{2}}\right) ^{2}\right] \nonumber \\&+BrM^{2}A_{2}\left( 1+ \frac{\partial \psi }{\partial y}\right) ^{2}\bigg \}^{2}dy, \end{aligned}$$

46 $$\begin{aligned} Err_{f}(N)= & {} \int ^{h}_{0}\left[ \frac{1}{Sc}\frac{\partial ^{2}f}{\partial y^{2}}-Kf\left( 1-f\right) ^{2}\right] ^2dy. \end{aligned}$$

Here *N* is the order of approximations. Substituting various order computational results into the above equations, the corresponding errors can be calculated, as illustrated in Table 4. It shows that computational errors reduces quickly as the computational order increases. The physical parameters used here are $d=0.4$, $x=0.1$, $\Theta = 1.2$, $W_e=0.05$, $\omega =0.03$, $n=0.2$, $R_{1}=0.3$, $Br=1$, $M=0.5$, $Sc=0.5$, $K_{s}=0.5$, $K=0.2$. This guarantees the convergence of the solutions and the 30th order computations are good enough to draw an analysis.

**Table 4:** The maximum calculated error *E*(*N*).

Order	$E_{\psi }$	$E_{\theta }$	$E_{f}$	$E_{\psi }$	$E_{\theta }$	$E_{f}$
	$\phi _{hnf}=0.025$			$\phi _{hnf}=0.05$		
10	1.03E−17	1.10E−19	1.07E−06	2.09E−17	2.23E−19	1.07E−06
20	4.29E−30	8.95E−30	1.37E−10	4.52E−30	8.24E−30	1.37E−10
30	5.23E−33	7.24E−32	7.32E−14	7.12E−33	7.56E−32	7.32E−14

## Entropy generation minimization

Basically, entropy is used to measure the disorder. To minimize the entropy generation, the mathematically volumetric entropy generation of hybrid nanofluid modeled by Ahmed et al.^57^ is adopted, which is written as

47 $$\begin{aligned} E_{gen}= & {} \frac{1}{T _{0}^2}\left[ k\left( \frac{\partial {\bar{T}}}{\partial {\bar{Y}}}\right) ^2+16\frac{{T}_{1}^{3}\sigma ^{*}}{3k^{*}}\left( \frac{\partial {{\bar{T}}}}{\partial {\bar{Y}}}\right) ^2 \right] +\frac{\sigma B_{0}^{2} {{\bar{U}}}^2}{T_{0}}+\frac{\Phi }{T_{0}}, \end{aligned}$$

where

48 $$\begin{aligned} \Phi =S_{{\bar{X}}{\bar{X}}}\frac{\partial {\bar{U}}}{\partial {\bar{X}}}+S_{{\bar{Y}}{\bar{Y}}}\frac{\partial {\bar{V}}}{\partial {\bar{Y}}}+S_{{\bar{X}}{\bar{Y}}}\left( \frac{\partial {\bar{U}}}{\partial {\bar{Y}}}+\frac{\partial {\bar{V}}}{\partial {\bar{X}}}\right) . \end{aligned}$$

On the right hand side of the expression (47), the first term represents the entropy generation due to the heat transfer and thermal radiation effect (also known as the heat transfer irreversibility ($H_{TI}$)), the second one is caused by the magnetic field (also called as the magnetic field irreversibility ($M_{FI}$)), the last term is the entropy generation due to the irreversibility of viscous dissipation (also known as the fluid friction irreversibility ($F_{FI}$)).

On the other hand, the characteristic entropy generation is defined as

49 $$\begin{aligned} E_{G}= \frac{k(T_{1}-T_{0})^2}{a^2}. \end{aligned}$$

The entropy generation which refers to the ratio of the volumetric entropy generation to the characteristic entropy generation rate, is therefore defined as

50 $$\begin{aligned} S_{G}= & {} \frac{E_{gen}}{E_{G}}= (A_{4}+R_{1})\frac{\partial ^{2}\theta }{\partial y^{2}}+\frac{Br}{\Omega }\left( \frac{\partial ^{2}\psi }{\partial y^{2}}\right) ^{2}\left[ 1+\frac{n-1}{2}W_e^{2}\left( \frac{ \partial ^{2}\psi }{\partial y^{2}}\right) ^{2}\right] \nonumber \\&+\frac{BrM^{2}A_{2}}{\Omega } \left( 1+\frac{\partial \psi }{\partial y}\right) ^{2}, \end{aligned}$$

where $\Omega =(T_{1}-T_{0})/{T_{0}}$ is the characteristic temperature difference ratio.

Moreover, the Bejan number $S_{B}$, namely, the relative entropy generation minimization, is defined as the ratio of the entropy generation due to heat transfer to the total entropy. It can be defined, based on the work of Das et al.^56^, as

51 $$\begin{aligned} S_{B}=\frac{H_{TI}}{H_{TI}+F_{FI}+M_{FI}}. \end{aligned}$$

It is worth mentioned that the range of Bejan number lies between 0 to 1 (refer to Shukla et al.^58^). When $S_{B}=0$, it is the limit at which the irreversibility due to viscous dissipation effect is dominated. When $S_{B}=0.5$, it means that the heat transfer irreversibility is dominant. Whereas $S_{B}=1$, it defines the case that the heat transfer irreversibility and the total entropy generation is equal.

## Result analysis

In this section, the influences of important physical emerging parameters on the flow behavior, heat transfer, homogeneous/heterogeneous and entropy are presented and analyzed.

**Figure 4:**
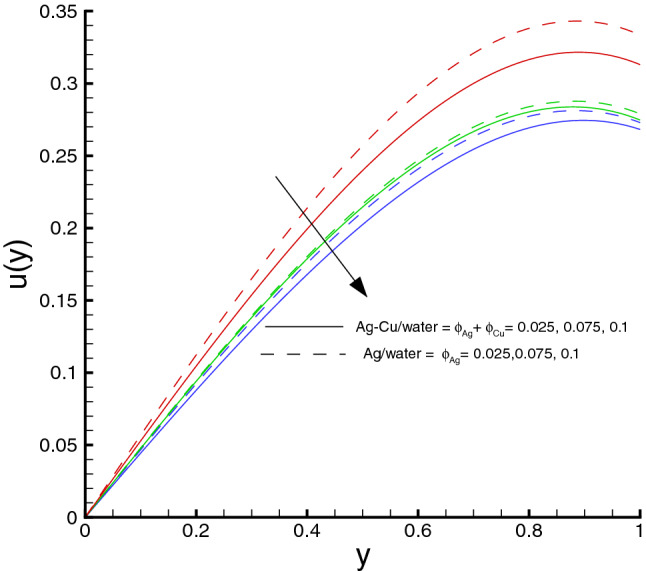
Velocity distribution against $\phi$, when $d=0.4, x=0.1, \Theta = 1.2, We=0.05, \omega =0.03, n=0.2, R_{1}=0.3, Br=1, M=0.5$.

The behaviour of the velocity field *u*(*y*) due to the effects of the volume concentration $\phi$, the Hartman number *M* and the Weissenberg number $W_e$ are explored through Fig. 4 and Table 5, respectively. Clearly, from Eq. (33), it is known that the nanomaterial volume fraction $\phi$ is an influential parameter for the flow field. The increase in the nanomaterial volume fraction causes the decrease of the velocity, as shown in Fig. 4. This can be explained from the definition of the viscosity of nanofluid (or the hybrid nanofluid) denoted in Table 2, in which it is readily to show that the viscosity increases by increasing the nanomaterial volume fraction, as a result the enhanced frictional force leads to the flow resistance and thus the velocity of fluid decreases. Note that here the scaling $y^{1/2} u(y)$ is used to replace *u*(*y*) for better distinction of those velocity profiles. As shown in Table 5, the increase in the Hartman number *M* results in the decrease of the longitudinal velocity at the center line of the channel. Physically, the Lorentz force enhances with the increase of *M*, which imparts additional momentum into the boundary layer. The boundary-layer thickness is therefore reduced, which, in turn, increases the surface shear stresses. On the other hand, it is shown in the table that the Weissenberg number $W_e$ exhibits similar influence on *u*(0) as *M* does, which also leads to that the additional momentum is added into the boundary layer. The surface shear stresses increase accordingly. We also notice that the decreasing trend is more distinguished in the hybrid nanofluid case as compared to the conventional nanofluid one. This indicates that more expensive or complicated nanofluids could be replaced by cheaper and simpler hybrid nanofluids with better heat transfer performance.

**Table 5:** Longitudinal velocity at the center line of channel for different parameters when $d=0.4$, $x=0.1$, $\Theta = 1.2$, $Br=1.0$, $R_{1}=0.3$, $\omega =0.03$, $\phi _{Ag}=\phi _{Ag}+\phi _{Cu}=0.05+0.05=0.1$ and $n=0.2$.

*M*	$W_e$	*u*(0) of water	*u*(0) of Ag/water	*u*(0) of Ag–Cu/water
0	0.05	0.725469	0.725469	0.725469
0.5		0.713086	0.709184	0.707914
1.0		0.679076	0.665659	0.661406
1.5		0.631103	0.607129	0.599803
0.5	0.01	0.714282	0.710399	0.709135
	0.05	0.713086	0.709184	0.707914
	0.1	0.709228	0.705265	0.703975
	0.2	0.691449	0.687148	0.685748

**Figure 5:**
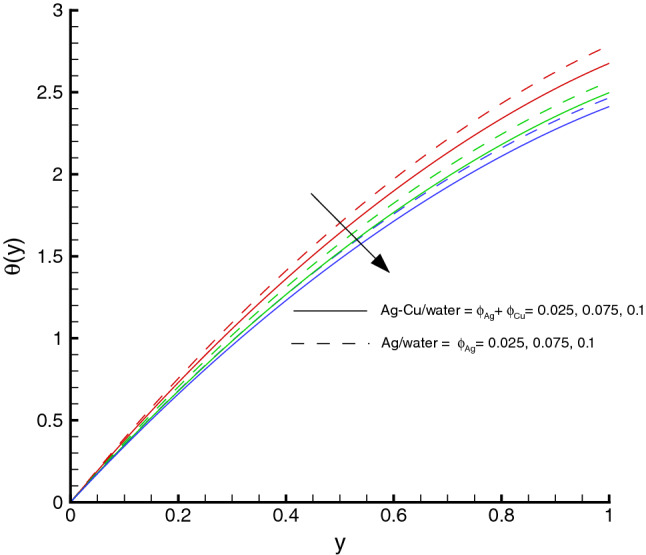
Temperature distribution against $\phi$, when $d=0.4, x=0.1, \Theta = 1.2, We=0.05, \omega =0.03, n=0.2, R_{1}=0.3, Br=1, M=0.5$.

**Figure 6:**
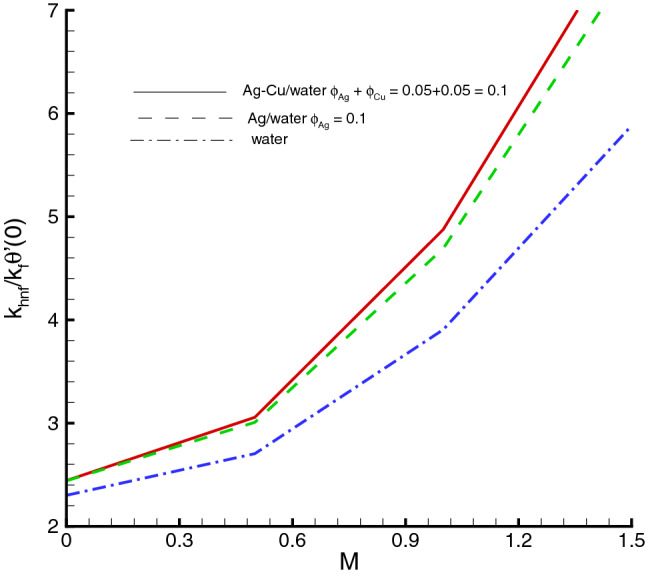
Heat transfer at the center of channel against *M*, when $d=0.4, x=0.1, \Theta = 1.2, We=0.05, \omega =0.03, n=0.2, R_{1}=0.3, Br=1$.

**Figure 7:**
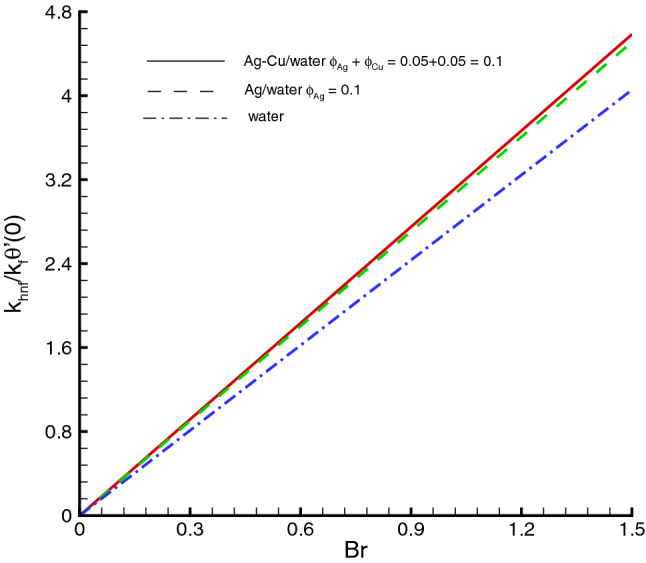
Heat transfer at the center of channel against *Br*, when $d=0.4, x=0.1, \Theta = 1.2, We=0.05, \omega =0.03, n=0.2, R_{1}=0.3,M=0.5$.

**Figure 8:**
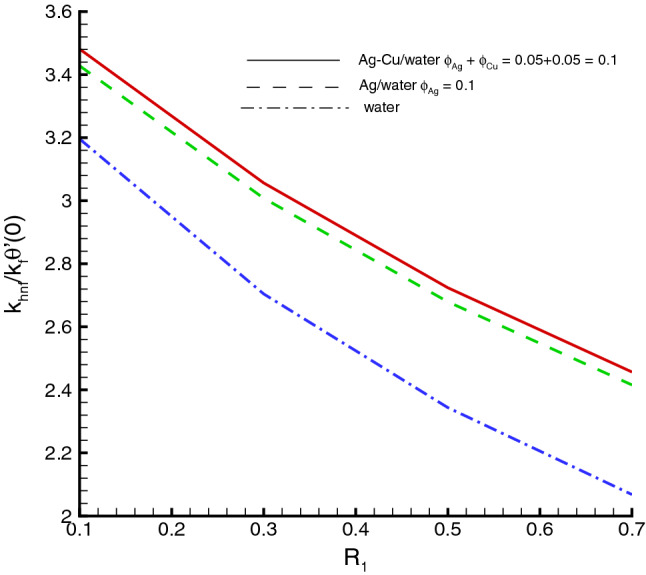
Heat transfer at the center of channel against $R_{1}$, when $d=0.4, x=0.1, \Theta = 1.2, We=0.05, \omega =0.03, n=0.2, Br=1, M=0.5$.

**Figure 9:**
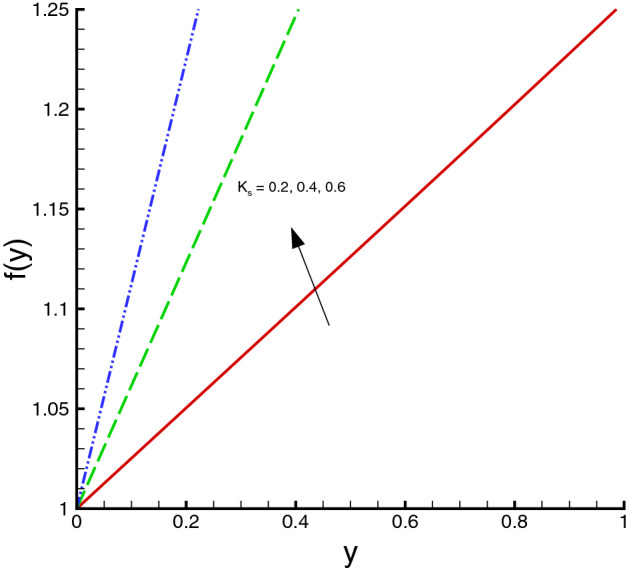
Homogeneous/heterogeneous distribution against $K_{s}$, when $d=0.4, x=0.1, \Theta = 1.2, Sc=0.5, K=0.2$.

**Figure 10:**
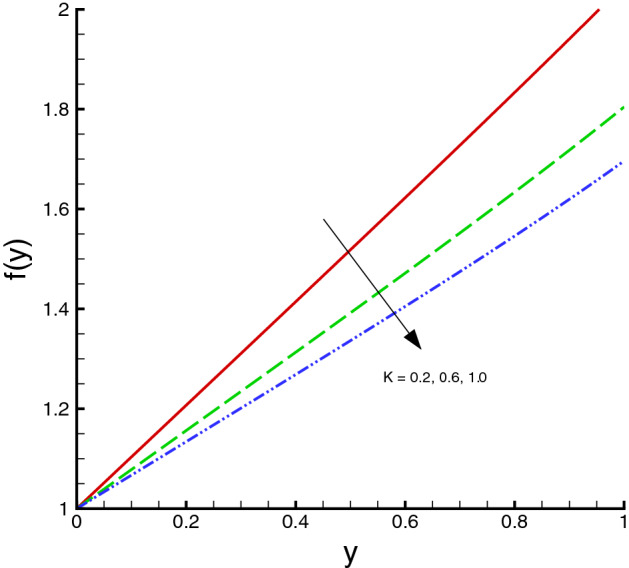
Homogeneous/heterogeneous distribution against *K*, when $d=0.4, x=0.1, \Theta = 1.2, Sc=0.5, K_{s}=0.5$.

**Figure 11:**
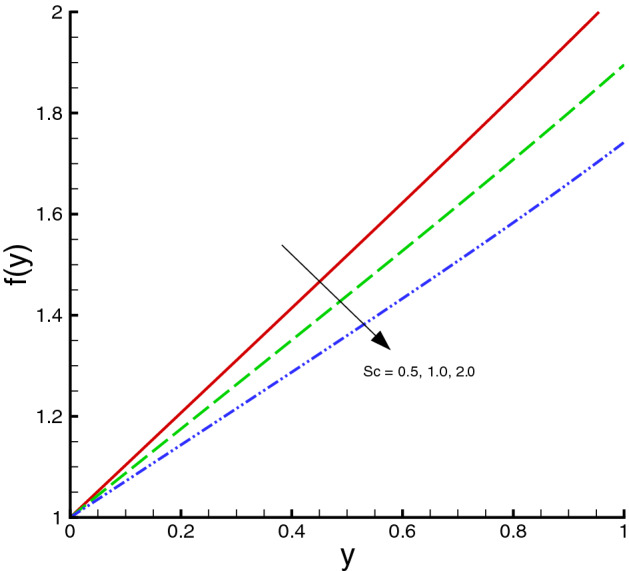
Homogeneous/heterogeneous distribution against *Sc*, when $d=0.4, x=0.1, \Theta = 1.2, K_{s}=0.5, K=0.2$.

The nanomaterials volume fraction has effect on the temperature distribution. This can be observed through Eqs. (33) and (34) in which the nanoparticles associated parameters are involved. As shown in Fig. 5, the increase in $\phi$ yields the reduction in temperature of fluid. The reason is that the thermal conductivity of the regular fluid enhances by adding nanoparticles to the base fluid and making the nanofluid or hybrid suspension. Thus, this higher thermal conductivity possesses a positive effect on the heat transfer performance and reduces the temperature of fluid. This observation also shows that silver-copper nanoparticles are good in used as coolants in numerous engineering and medical applications. Such change is distinct for the *Ag*/*water* nanofluid as compared to the *Ag*–*Cu*/*water* hybrid nanofluid. The influences of different physical parameters on the temperature distribution on the peristaltic wall are presented in Table 6. It is observed that the pure water possesses high temperature as compared to the nanofluid and the hybrid nanofluid. It is justified by the fact that the nanoparticles can be used as coolants. A development in temperature is obtained for larger *M* and *Br*. This effect is quite obvious because of the applied magnetic field and the dominant factor of viscous dissipation effect for larger Brinkman number. While a decreasing behavior is observed for the temperature distribution under the effect of the thermal radiation. Because the increment in $R_{1}$ means the decrease in the mean absorption parameter, which ultimately results in the temperature decrease. The heat transfer rate at the center line of the channel is illustrated in Figs. 6, 7 and 8, respectively. Here the comparison is made among the regular fluid, the nanofluid and the hybrid nanofluid. Fig. 6 exhibits the variation of heat transfer rate against *M*. Clearly, the heat transfer rate enhances significantly as *M* increases. It justifies that the enlarged Hartman number specifies the retarding body force that slackens the fluid motion as a result the temperature of fluid increases and consequently heat transfer rate enhances. The effect of *Br* on the heat transfer rate is shown in Fig. 7. It is clearly seen in this figure that the heat transfer rate of silver–copper–water hybrid nanofluid is the highest, then the silver–water nanofluid and finally the pure water. This is due to the hybrid nanofluids have better thermal conductivity, which accelerate the heat transfer between the solid boundary and the ambient fluid. Hence, make use of hybrid nanofluid instead of the nanofluid is more valuable in thermal management systems. Decreasing behavior of the heat transfer rate against $R_{1}$ is depicted in Fig. 8. Since the increase in the thermal radiation minimizes the mean absorption parameter, consequently the heat losses and thus the heat transfer rate weakens. Such decrease trend is more distinct in the pure water case as compared to the nanofluid and the hybrid nanofluid cases. Figs.9, 10 and 11 demonstrate influences of the homogeneous and heterogeneous reaction parameters, as well as the Schmidt number on distribution of the chemical species. As shown in Fig. 9, the increase in the heterogeneous reaction parameter $K_{s}$ is helpful to increase the concentration of the chemical species *A* since the acceleration of the heterogeneous reaction means more chemical species *A* involve into the chemical reaction on the chemical front, which, in turn, enhances the concentration of the chemical species *A*. Influence of the homogeneous reaction parameter *K* on the concentration of the chemical species is described in Fig. 10. As the homogeneous chemical reaction becomes stronger (*K* increases), more species *A* are consumed in the bulk, which leads to a decrease trend of the concentration of the chemical species *A*. From Eq. (37), it is known that *Sc* is also a key factor to affect the distribution of the chemical reactant. This influence is shown in Fig. 11, in which the concentration of species *A* decreases as *Sc* evolves. The reason is that the Schmidt number *Sc* is ratio of momentum to mass diffusivities, as a result the increase in *Sc* results in the reduction of this concentration.

**Table 6:** Temperature distribution on the peristaltic wall with different influential parameters when $d=0.4$, $x=0.1$, $\Theta = 1.2$, $W_e=0.05$, $\omega =0.03$, $\phi _{Ag}=\phi _{Ag}+\phi _{Cu}=0.05+0.05=0.1$ and $n=0.2$.

*M*	*Br*	$R_{1}$	$\theta (h)$ of water	$\theta (h)$ of Ag/water	$\theta (h)$ of Ag–Cu/water
0	1.0	0.3	2.284990	1.812512	1.820429
0.5			2.475495	2.029223	2.021767
1.0			3.056466	2.693973	2.638511
1.5			4.050345	3.838454	3.698867
0.5	0.1		0.247549	0.202922	0.202176
	0.5		1.237747	1.014612	1.010883
	1.0		2.475495	2.029223	2.021767
	1.5		3.713243	3.043835	3.032651
0.5	1.0	0.1	2.925585	2.311279	2.304186
		0.3	2.475495	2.029223	2.021767
		0.5	2.145429	1.808521	1.801020
		0.7	1.893025	1.631118	1.623733

**Figure 12:**
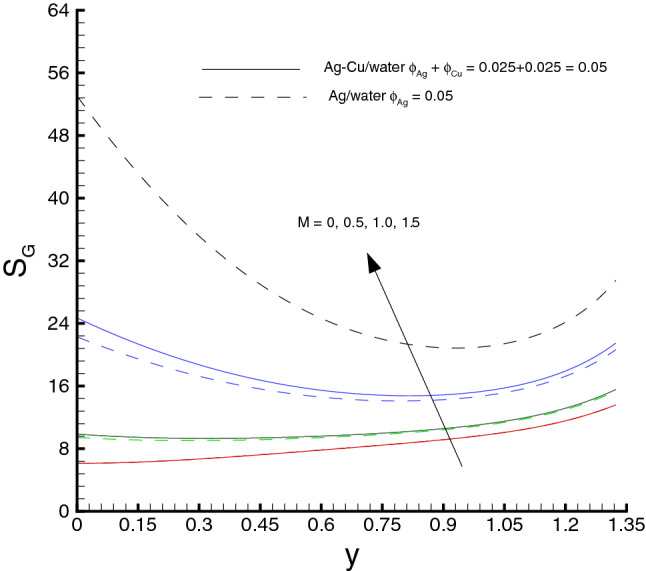
Entropy generation distribution against *M*, when $d=0.4, x=0.1,\Theta = 1.2, We=0.05, \omega =0.03, n=0.2, R_{1}=0.3, Br=1$.

**Figure 13:**
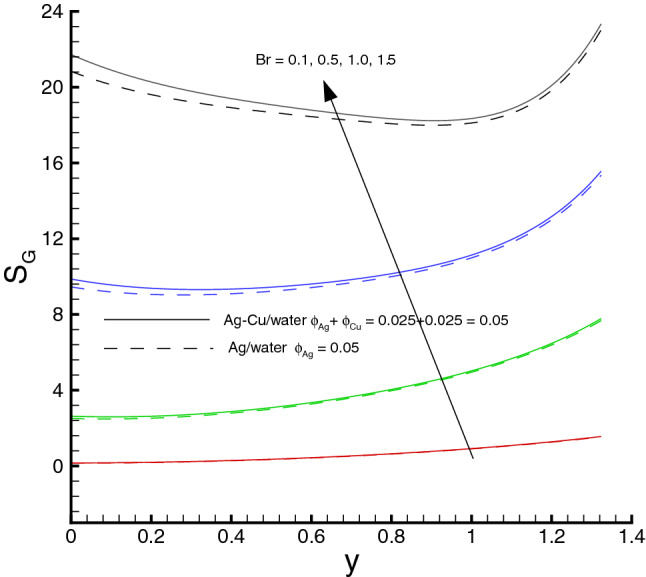
Entropy generation distribution against *Br*, when $d=0.4, x=0.1,\Theta = 1.2, We=0.05, \omega =0.03, n=0.2, R_{1}=0.3, M=0.5$.

**Figure 14:**
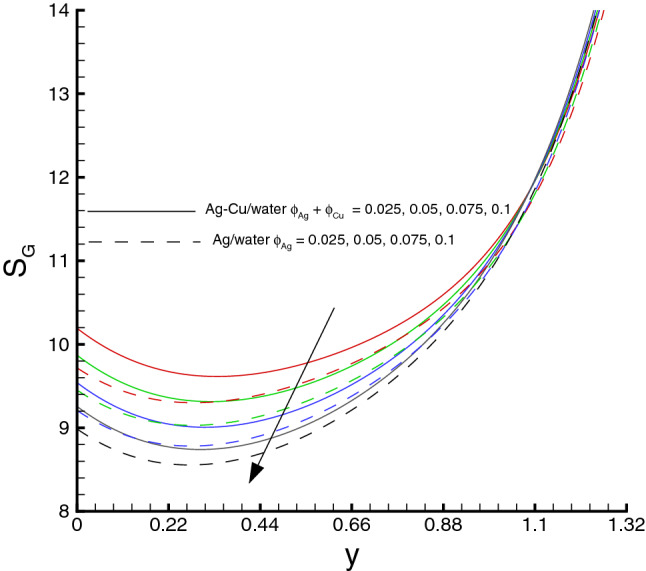
Entropy generation distribution against $\phi$, when $d=0.4, x=0.1,\Theta = 1.2, We=0.05, \omega =0.03, n=0.2, R_{1}=0.3, Br=1, M=0.5$.

**Figure 15:**
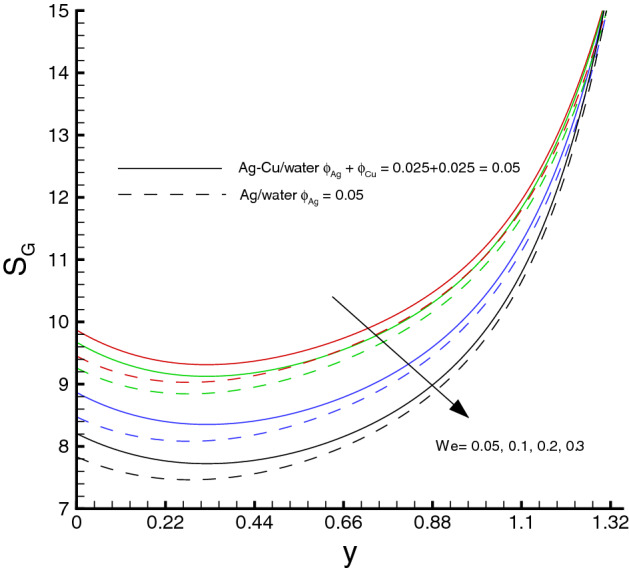
Entropy generation distribution against *We*, when $d=0.4, x=0.1,\Theta = 1.2, M=0.5, \omega =0.03, n=0.2, R_{1}=0.3, Br=1$.

**Figure 16:**
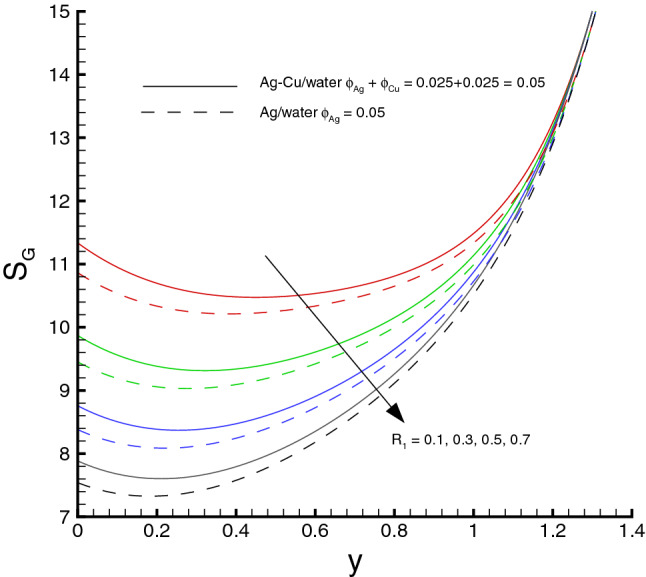
Entropy generation distribution against $R_{1}$, when $d=0.4, x=0.1,\Theta = 1.2, We=0.05, \omega =0.03, n=0.2, M=0.5, Br=1$.

**Figure 17:**
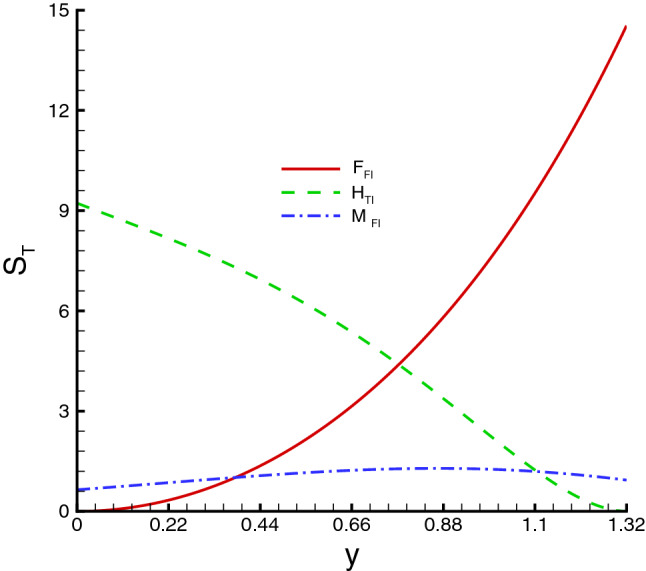
Effect of physical parameters $d=0.4, x=0.1,\Theta = 1.2, We=0.05, \omega =0.03, n=0.2, M=0.5, Br=1, R_{1}=0.3, \phi _{Ag}=\phi _{Ag}+\phi _{Cu}=0.025+0.025=0.05$ on relative entropy generation minimization.

Variations of various emerging parameters on entropy generation are visualized through Figs. 12, 13, 14, 15 and 16. In Fig. 12, the effect of *M* on the entropy generation is illustrated. It is clearly observed that the increase in *M* has a tendency for enhancement of the entropy generation because the strength of magnetic field increases, consequently the temperature of fluid upsurge and as a result the entropy generation increases. Similarly, an increase in the entropy generation for growing values of *Br* is presented in Fig. 13. It is due to larger values of Brinkman number intensifies the fluid friction and heat transfer rates of the fluid, thus, entropy generation number significantly increases with growing values of *Br*. It is also noticed that the enrichment in entropy generation for *Ag*-*Cu*/*water* hybrid nanofluid is more pronounced then the *Ag*/*water* nanofluid. A decreasing trend in entropy generation is perceived as the nanoparticles volume fraction increases, as shown in Fig. 14. It is due to the increase in viscosity by adding nanoparticles to the base fluid. Figs. 15 and 16 exhibit the influences of $W_e$ and $R_{1}$ on the entropy generation. It is seen that the entropy generation reduces as either of them increases due to loss of heat. It is revealed from these figures that the decreasing rate is faster for the *Ag*/*water* nanofluid as compared to the *Ag*–*Cu*/*water* hybrid nanofluid. Fig. 17 shows the involvement of various sources of entropy generation in the peristaltic channel. Apparently irreversibility due to the viscous dissipation ($F_{FI}$) approaches zero at the inlet of the channel, while it has a dominant effect at the wall of the channel. Furthermore entropy generation because of thermal diffusion ($H_{TI}$) is a major factor in the vicinity at the inlet of the channel, whereas it has a decreasing behavior towards the wall of the channel. The third factor of entropy generation due the applied magnetic filed effect ($M_{FI}$) exerts a minimum impact at the wall surface.

## Conclusions

The steady, laminar and incompressible electrical conducting peristaltic flow of a *Ag*/water nanofluid and/or a Ag–Cu/water hybrid nanofluid in a symmetric channel has been inspected in detail. Because both nanofluids and hybrid nanofluids with applied magnetic field are used in cancer therapy and found to be more adhesive for tumor cells than non-malignant cells. Thermodynamic optimization aspect has also been discussed through entropy generation analysis. In summary, the novel aspects of presented flow analysis are:

1. Decrease in velocity profile is revealed for growing values of the Weissenberg parameter.
2. Reduction in velocity and temperature field is discovered for both conventional nanofluid and hybrid nanofluid via nanoparticle volume fraction.
3. Hartman number *M* enhances the heat transfer rate. Such observation shows that hybrid nanofluid has high temperature. Which authenticates that the hybrid nanofluids may helpful to increase the physical properties of fluid.
4. Variation of thermal radiation parameter results in the decrease of the heat transfer rate.
5. Opposite response of concentration field is noticed for heterogeneous and homogeneous reaction parameters respectively.
6. Entropy generation is an increasing function of the Brinkman number. Such increment is higher for Ag–Cu/water hybrid nanofluid, then followed by *Ag*/*water* nanofluid.

## Nomenclature

*V*: Velocity of the nanofluid
*B*: Magnetic field
*J*: Current density
*P*: Pressure
*T*: Temperature
$$c_{p}$$: Heat capacity
$$\lambda $$: Wave length
*k*: Thermal conductivity
2*a*: Width of the channel
${\bar{\alpha }}$: Concentration of chemical species A
${\bar{\beta }}$: Concentration of chemical species B
*b*: Amplitude of the peristaltic wave
$k_{c}$: Homogeneous reaction coefficient
$k_{s}$: Heterogeneous reaction coefficient
$D_{A}$: Diffusion coefficient of chemical species A
$D_{B}$: Diffusion coefficient of chemical species B
$c_{p}$: Specific heat at constant pressure
*Sc*: Schmidt number
*Re*: Reynold number
*M*: Hartman number
*Br*: Brinkman number
$R_{1}$: Thermal radiation parameter
*We*: Weissenberg number
$$\rho $$: Density
$$\eta _{0}$$: Viscosity
$\epsilon$: Homogeneous reaction ratio
$$\sigma $$: Electric conductivity
$\delta$: Dimensionless wave number
*F*: Dimensionless flow rate
$\psi$: Dimensionless stream function
$\sigma ^*$: Stefan-Boltzman constant
$k^*$: Mean absorption coefficient
$k^*$: Mean absorption coefficient
$$\theta $$: Dimensionless temperature
$K_{s}$: Dimensionless homogeneous reaction parameter
*K*: Dimensionless heterogeneous reaction parameter
$\omega$: Dimensionless velocity slip parameter
$S_{G}$: Dimensionless entropy generation rate
$\Omega$: Characteristic temperature difference ratio
*f*: Dimensionless concentration of chemical species A
*g*: Dimensionless concentration of chemical species B
*Pr*: Prandtle number
